# Research on Bitter Peptides in the Field of Bioinformatics: A Comprehensive Review

**DOI:** 10.3390/ijms25189844

**Published:** 2024-09-12

**Authors:** Shanghua Liu, Tianyu Shi, Junwen Yu, Rui Li, Hao Lin, Kejun Deng

**Affiliations:** School of Life Science and Technology, Center for Informational Biology, University of Electronic Science and Technology of China, Chengdu 610054, China; 202321140418@std.uestc.edu.cn (S.L.); 202221140537@std.uestc.edu.cn (T.S.); 202322140206@std.uestc.edu.cn (J.Y.); 202321140412@std.uestc.edu.cn (R.L.)

**Keywords:** bitter peptides, bitterness mechanism, database, predictive models, bioinformatics

## Abstract

Bitter peptides are small molecular peptides produced by the hydrolysis of proteins under acidic, alkaline, or enzymatic conditions. These peptides can enhance food flavor and offer various health benefits, with attributes such as antihypertensive, antidiabetic, antioxidant, antibacterial, and immune-regulating properties. They show significant potential in the development of functional foods and the prevention and treatment of diseases. This review introduces the diverse sources of bitter peptides and discusses the mechanisms of bitterness generation and their physiological functions in the taste system. Additionally, it emphasizes the application of bioinformatics in bitter peptide research, including the establishment and improvement of bitter peptide databases, the use of quantitative structure–activity relationship (QSAR) models to predict bitterness thresholds, and the latest advancements in classification prediction models built using machine learning and deep learning algorithms for bitter peptide identification. Future research directions include enhancing databases, diversifying models, and applying generative models to advance bitter peptide research towards deepening and discovering more practical applications.

## 1. Introduction

In vertebrates, taste is one of the fundamental physiological senses, helping organisms to select palatable foods and identify toxic and nutritious substances [1]. For a long time, the mainstream view of the scientific community has been that human taste can sense the five basic tastes of sour, sweet, bitter, salty, and fresh. A sixth taste, named the ammonium chloride taste, has been reported recently [2]. Bitterness typically arises from various substances such as polyphenols and alkaloids in food. Bitter peptides are produced from the breakdown of proteins under acidic, alkaline, or enzymatic conditions [3]. Most of these peptides are typically composed of no more than eight amino acids, and few contain more than ten. However, bitter peptides containing up to 39 amino acids have been described [4]. In food science, the presence of bitter peptides significantly impacts the taste perception of food products. Because of their unique amino acid composition and sequence structure, these short-chain peptides provide some benefits in humans, including antihypertensive, antidiabetic, antioxidant, antibacterial, and immunological effects [5]. Considering the bioactivity of bitter peptides, integrating them into everyday foods and beverages can lead to the development of functional foods that are both tasty and health-promoting, thus enhancing dietary quality and public health [6,7]. In biomedical research, bitter peptides have shown potential involvement in regulating the secretion of digestive enzymes, promoting gastrointestinal motility, and affecting the defensive mechanisms of the respiratory tract [8,9,10].

As early as 1953, Raadsveld et al. isolated bitter compounds from Gouda cheese and identified them as peptides, using human sensory evaluation to verify their bitterness [11]. Later, experiments by Murray and Baker found that treating casein with various proteases led to the accumulation of bitter peptides, and they successfully isolated and identified a specific bitter peptide [12,13,14]. Before the rise of bioinformatics, the identification of bitter peptides relied on a range of intricate experimental methods, each with its characteristics and limitations. (1) High-performance liquid chromatography (HPLC) and mass spectrometry (MS) offered precise separation and mass identification, but were costly and complex to operate [15]. (2) Synchrotron infrared and circular dichroism spectroscopy were used to explore peptide structures, providing deep insights into bitterness mechanisms; however, these techniques demanded a high sample quality and were technically complex [16,17,18,19,20]. (3) Amino acid sequence analysis provided the detailed identification of peptide structures, but was time-consuming and cumbersome [21]. (4) Cell-based sensory receptor assays allowed for direct evaluations of the bioactivity of bitter peptides [22], while animal behavioral experiments assessed bitterness through animal feeding preferences [23]. (5) Human sensory evaluation directly reflected consumer experience, but suffered from significant subjectivity. In summary, these experimental methods uniquely contributed to revealing the characteristics of bitter peptides, but faced challenges such as cost, efficiency, and implementation difficulty [24].

In recent years, with the rapid development of bioinformatics, significant progress has been made in bitter peptide research. BitterDB, databases containing bitter peptide information, have provided resources for research and development [25]. Researchers have developed various algorithms to predict and identify bitter peptides based on sequence using machine learning and deep learning techniques, significantly improving prediction accuracy [26]. Bioinformatics tools are widely applied to predict bitterness thresholds based on sequence [27,28,29,30]. Through molecular dynamics simulations and structure predictions, researchers can gain a deep understanding of the secondary structures of bitter peptides, revealing their functional properties [31,32,33,34]. These studies provide a solid theoretical foundation and technical support for bitter peptides in food science and drug development [35,36,37,38].

**Figure 1:**
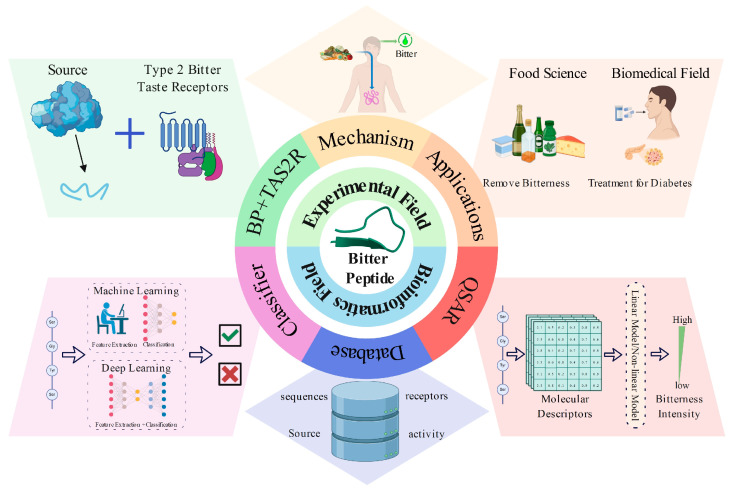
A schematic diagram of the content covered in this review. It primarily includes two sections: experimental field and bioinformatics field. In the experimental section, we introduce bitter peptides (BPs) and taste receptors type 2 (TAS2Rs), the mechanisms of bitterness generation, and their applications. In the bioinformatics section, we discuss bitter peptide databases, QSAR models, and classification models.

This review introduces the properties of bitter peptides, the mechanisms of bitterness generation, and their physiological functions and applications, highlighting research in bioinformatics related to bitter peptides, including bitter peptide databases, QSAR models, and predictive models (Figure 1). Furthermore, it provides an outlook on future research directions.

## 2. Bitter Peptides

A bitter peptide is a flavor peptide produced by the hydrolysis of proteins in food through proteolytic enzymes. Flavor peptides are oligopeptides with a molecular mass of less than 3000 u, which can bind to corresponding taste receptors on the tongue to present characteristic flavors [39,40]. In food, based on the flavor-imparting action of flavor peptides, they can be divided into flavor precursor peptides and characteristic flavor peptides. Characteristic flavor peptides, including sweet peptides, sour peptides, bitter peptides, salty peptides, and umami peptides, have specific effects [41]. This review will focus on bitter peptides.

### 2.1. Sources of Bitter Peptides

Bitter peptides are widely found in everyday foods and primarily originate from three sources: (1) natural sources, which are peptides directly extracted from natural foods such as meats and dairy products; (2) fermentation production, where the presence of fermenting microbes in cheese, fermented fish products, and bean products produces bitter peptides; and (3) enzymatic production, which uses enzymes to process protein from raw materials (such as milk protein, soy protein, etc.) in industrial or laboratory settings, also resulting in the formation of bitter peptides [42,43,44,45,46]. These peptides are essentially hydrolyzed protein products. The exposure of hydrophobic amino acids within these peptides often stimulates the taste buds, causing bitterness. Generally, the more hydrophobic amino acids are exposed, the stronger this bitterness [47,48,49,50,51]. Additionally, the length of the peptide chain, the overall hydrophobicity, the sequence, and the amino acid composition also significantly affect bitterness [52].

### 2.2. Extraction of Bitter Peptides

Compared to the sources of bitter peptides, how to extract them is more important. There are different extraction methods for fermented products and non-fermented products. (1) In fermented products, proteins are enzymatically hydrolyzed into short peptides during fermentation. So, these peptides can be directly extracted. The typical extraction of peptides involves the following steps: first, the fermented product (such as cheese, soy sauce, or yogurt) is homogenized or ground to increase its surface area. Next, an appropriate solvent (such as water or a 50% ethanol solution) is used to extract the peptides, typically at 1:5 (sample, *w*/*v*). The extraction solution is stirred at 4 °C for 1–2 h to ensure thorough dissolution of the peptides. The mixture is centrifuged (10,000 rpm, 15 min) and filtered through a 0.22 µm membrane to remove solid impurities, yielding a short peptides solution [53]. (2) In non-fermented products, protein extraction is required first, followed by hydrolysis to produce short peptides. The extraction steps include grinding the sample (such as meat or grains) and dissolving the proteins in 0.1 M phosphate buffer (pH 7.0) at a ratio of 1:10 (sample, *w*/*v*). The solution is stirred at 4 °C for 2 h and sonicated (40 kHz, 10 min) to assist with extraction. The extracted sample is centrifuged (10,000 rpm, 20 min) and filtered (0.22 µm membrane) to obtain a purified protein solution. Subsequently, an appropriate enzyme (such as trypsin, at an enzyme-to-protein ratio of 1:100, *w*/*w*) is selected for hydrolysis at 37 °C for 4–24 h, or 6 M hydrochloric acid is added to hydrolysis at 110 °C for 24 h. The hydrolyzed product is then centrifuged, dialyzed, and concentrated to obtain short peptides [54,55].

The resulting supernatant obtained through the above methods can be directly analyzed using experimental techniques such as high-performance liquid chromatography (HPLC) and mass spectrometry (MS) or subjected to sequencing and bioinformatics methods (as discussed in Section 3) for further analysis to identify and characterize the composition and properties of bitter peptides.

### 2.3. Chemical and Physiological Mechanisms of Bitter Taste Perception

Understanding the diverse sources of bitter peptides has provided valuable insights into the factors that contribute to their distinct bitter taste. The main reason for the bitterness produced by protein hydrolysis is the formation of bitter peptides. The factors influencing the bitterness thresholds in bitter peptides include the hydrophobicity of the peptides, their sequence and number of amino acids, their composition, and the length and molecular weight of the peptide chains [52,56]. Bitter peptides generally contain several amino acids with hydrophobic side chains (such as phenylalanine, tyrosine, and tryptophan) [41,42,57]. In 1971, Ney et al. proposed the Q rule, primarily used to describe and predict the bitterness level of peptides by calculating the frequency of hydrophobic amino acid residues to obtain a Q value. Peptides with a Q value less than 1300 are not bitter, while those with a Q value greater than 1400 are bitter [58]. To date, the Q rule is a parameter still used to assess the relationship between the hydrophobicity of peptide chains and bitterness [46,59,60].

While the chemical properties of bitter peptides explain their origins, the physiological mechanisms by which animals perceive this taste are equally important. Exploring how the gustatory system processes these peptides sheds light on the complex interaction between their molecular structure and taste perception. The gustatory system is a complex physiological structure with taste buds associated with the nerve and brain structures. The tongue, the organ of the gustatory system, is covered with papillae, each harboring taste buds—the basic units of taste [61,62]. Taste buds contain various types of gustatory cells, such as Type I, II, III, and IV, which convert food stimuli into electrical signals that are transmitted to the brain to produce taste [63,64,65]. Specifically, bitter taste receptors (taste receptors type 2, TAS2Rs), part of the G protein-coupled receptor family, are predominantly expressed in Type II cells. They recognize ligands and transmit signals through a seven-transmembrane region and intracellular and extracellular loop structures. These receptors, which are expressed in diabetes in the oral cavity and extra-oral tissues, such as the gastrointestinal tract, airways, brain, and testes, demonstrate the extensive and complex functionality of the gustatory system [44,66,67,68,69,70,71,72].

When a bitter peptide binds to a TAS2R, it induces a conformational change and follows the dissociation of the α-subunit from the β- and γ-subunits of gustducin. This dissociation of subunits marks the initiation of two distinct signaling pathways [73,74,75,76]: (1) In the first pathway, the β- and γ-subunits activate phospholipase C β2 isoform (PLCβ2), which cleaves phosphatidylinositol 4,5-bisphosphate (PIP2) into diacylglycerol (DAG) and inositol 1,4,5-trisphosphate (IP3). IP3 then enters the endoplasmic reticulum (ER), where it binds to the IP3 receptor (IP3R), causing the release of Ca^2+^ from the ER into the cytoplasm. These concentration increases in intracellular Ca^2+^ activate the sodium-selective transmembrane transporters transient receptor potential melastatin 4 and 5 (TrpM4/5), leading to cell membrane depolarization. Depolarization activates voltage-gated sodium channels (VGNCs), further accelerating the depolarization. Upon reaching an action potential (AP), the calcium homeostasis modulator 1/3 (CALHM1/3) channels and pannexin 1 channels are activated and allow adenosine triphosphate (ATP) to be transported from the cytoplasm to the intercellular space. ATP is taken up by incoming neurons via P2X purinergic receptors 2/3 (P2X2/P2X3), further propagating the signal. (2) The second pathway involves the α-subunit of gustducin. α-gustducin reduces cAMP levels by activating phosphodiesterase (PDE), which hydrolyzes cyclic adenosine monophosphate (cAMP). This reduction in cAMP may decrease intracellular cyclic nucleotide monophosphate (cNMP) levels, thereby regulating protein kinases and subsequently modulating ion activity within the cell. Alternatively, cNMPs might directly regulate cNMP-gated ion channels, leading to membrane depolarization and the release of neurotransmitters [77,78,79]. These cellular responses ultimately lead to the transmission of neural signals, with bitter information being relayed from the gustatory receptor cells through the facial nerve and the glossopharyngeal nerve to the solitary tract nucleus (NTS) in the brainstem, then to the parabrachial nucleus (PbN), and finally to the gustatory cortex in the thalamus and cerebral cortex [80,81,82].

These detailed signal transduction pathways reveal the neural basis of bitter taste perception and highlight the potential role of bitter peptides in regulating physiological functions such as digestion and respiration, providing new perspectives for treating diseases related to these processes.

### 2.4. Functions and Applications of Bitter Peptides

Having explored the intricate mechanisms by which animals perceive bitterness through the gustatory system, these bitter peptides might offer significant health benefits. Current research on bitter peptides primarily focuses on food science and biomedicine [83].

In food science, bitter peptides can significantly influence the taste profile of a food product, prompting researchers to focus on optimizing production processes to control their levels. This optimization often involves adjusting fine-tuned enzymatic hydrolysis conditions, such as the pH, temperature, and enzyme-to-substrate ratios, to minimize the formation of bitterness-contributing peptides. There is a bell-shaped relationship between the degree of hydrolysis (DH) and bitterness intensity; controlling the DH can significantly affect the sensory quality of protein hydrolysates [84,85,86,87]. Moreover, based on the bioactivity of bitter peptides, researchers can develop functional foods that are both tasty and beneficial for health by incorporating these functional peptides into everyday foods and beverages. Various encapsulation techniques, such as spray drying, liposomes, ionic gelation, freeze-drying, and double emulsion solvent evaporation, can enhance their stability, bioavailability, and consumer acceptance. These methods help to protect these bioactive peptides from degradation, control their release, and mask undesirable flavors, making them suitable for use in food products [88,89].

In biomedicine, bitter peptides exhibit a range of functions through their interactions with bitter receptors located in various tissues, including the oral cavity, gastrointestinal and respiratory systems, pancreatic and ovarian tissues, and associated malignant tumors [90]. These interactions can regulate digestive enzyme secretion, promote gastrointestinal motility, and modulate respiratory defense mechanisms [41,91]. Building on these properties, researchers have developed microcapsules to encapsulate bitter peptides and other functional peptides, effectively masking their bitterness, preserving their antioxidant activity, and specifically targeting gastrointestinal bitter receptors to enhance therapeutic efficacy [92].

Moreover, bitter peptides are valuable for their antihypertensive, antidiabetic, antioxidative, antibacterial, and immune regulation properties. (1) Antihypertensive: Bitter peptides regulate blood pressure by inhibiting angiotensin-converting enzyme (ACE) and renin, while also enhancing the endothelial nitric oxide synthase (eNOS) pathway to increase the nitric oxide (NO) levels in blood vessels, thereby promoting vasodilation [93]. One study demonstrated that hydrolysates of bitter apricot protein, such as the peptide RPPSEDEDQE, exhibited ACE inhibitory activity and zinc-ion-chelating abilities by forming hydrogen bonds and undergoing hydrophobic interactions at multiple active sites of ACE. In spontaneously hypertensive rats, these peptides significantly reduced their blood pressure, although zinc chelation diminished their antihypertensive effect, indicating their potential in hypertension management [94,95,96]. Interesting, Val-Pro-Pro (VPP) and Ile-Pro-Pro (IPP) are two tripeptides found in fermented dairy products, known for their antihypertensive bioactivity, which primarily lower blood pressure by inhibiting the activity of ACE [97]. (2) Antidiabetic: Bitter peptides have shown significant potential in managing diabetes through various mechanisms. Studies have shown that mcIRBP-19-BGE can span the 50th–68th residues, enhance the binding of insulin and insulin receptors (IRs), stimulate the phosphorylation of phosphoinositide-dependent kinase-1 (PDK1) and protein kinase B (PKB), and induce the expression of glucose transporter 4, thus promoting glucose clearance [98,99,100,101]. In a clinical setting, a study involving 142 diabetic patients demonstrated that bitter melon peptide (BMP) treatment significantly reduced their glycated hemoglobin (HbA1c) levels starting from the second month, underscoring its therapeutic potential [98]. Furthermore, Pan et al. used the Health Belief Model to study the factors affecting BMP intake among 292 type 2 diabetes patients in Taiwan. They found that perceived susceptibility to diabetes complications and perceived benefits were key motivators for BMP consumption, with self-efficacy significantly enhancing the likelihood of intake [102]. Broader research on bitter peptides in herbal remedies can improve insulin sensitivity and manage blood sugar by inhibiting glucose absorption and boosting insulin receptor activity [39,40,103,104,105]. (3) Antioxidative properties: Bitter peptides exert antioxidant effects through scavenging free radicals, neutralizing reactive oxygen species, chelating transition metal ions, forming a physical barrier at the oil–water interface, and so on [106]. Numerous studies have demonstrated the antioxidant activities of protein hydrolysates and peptides. For instance, Carrillo et al. reported that the bitter peptides in casein hydrolysates effectively inhibited lipid peroxidation in olive oil by scavenging free radicals and neutralizing reactive oxygen species, highlighting their significant antioxidant potential [107]. Similarly, Li et al. found that the bitter peptides in whey isolate hydrolysates reduced protein oxidation in carp surimi by maintaining protein solubility, preserving Ca-ATPase activity, and minimizing structural changes during frozen storage [108,109]. (4) Antibacterial: Bitter peptides have shown significant potential as natural antimicrobial agents in various food applications. Filho et al. found that incorporating hydrolyzed cottonseed protein, rich in bitter peptides, into alginate films effectively inhibited the growth of Staphylococcus aureus by disrupting bacterial cell membranes and interfering with microbial metabolism, making these films a promising choice for active food packaging [110]. Similarly, Dang et al. discovered that antimicrobial peptides, including bitter peptides isolated from the edible insect Musca domestica, inhibited the growth of *Staphylococcus aureus*, *Escherichia coli*, *Salmonella* spp., and *Listeria monocytogenes* in chilled pork, significantly extending its shelf life by increasing its bacterial membrane permeability and causing cell lysis [111]. Ren et al. also demonstrated that adding catfish bone hydrolysates rich in bitter peptides could effectively suppress microbial growth in catfish sausages, enhancing their safety and prolonging their shelf life during ambient temperature storage [112]. (5) Immune regulation: Bitter peptides have emerged as promising agents in modulating immune responses. Some plant-derived bitter peptides, such as lunasin and soy-derived peptides, have been demonstrated to stimulate natural killer (NK) cells and enhance the phagocytic activity of macrophages. These peptides exert their immunomodulatory effects by activating immune cells and promoting cytokine production, strengthening the body’s innate immune response and providing enhanced protection against infections and diseases [113,114,115,116].

Therefore, research on bitter peptides has enhanced our understanding of the causes and regulation of bitterness in food and provided a scientific basis for developing new therapeutic methods. With advancements in food science and biotechnology, further exploring the multifunctionality and potential applications of bitter peptides will become a challenging and valuable field of study.

## 3. Research on Bitter Peptides in Bioinformatics

Bitter peptides are known for their ability to interact with taste receptors type 2 (TAS2Rs) in the mouth [117,118]. Therefore, studying and characterizing their bitterness thresholds is crucial, as this plays a significant role in drug development and nutritional research [94,104]. Although experimental methods are considered to be reliable for characterizing peptide bitterness, they are often time-consuming and expensive. Machine learning (ML) methods, known for their convenience and efficiency, are increasingly attracting attention in bioinformatics.

Significant progress has been made in studying bitter peptides, providing new perspectives and methodologies for peptide research in bioinformatics. Firstly, databases related to bitter peptides have been developed to store and manage the vast information on bitter peptides and their characteristics, offering valuable resources for researchers. Secondly, the quantitative structure–activity relationship (QSAR) and classification models for bitter peptides continue to be refined. These models use machine learning techniques to predict the activity and stability of bitter peptides, significantly enhancing research efficiency. The current research provides inspiration and methodological references for the study of bitter peptides and deepens our understanding of the bioactivity of peptides overall. In the future, bioinformatics is expected to develop more innovative technologies for studying bitter peptides.

### 3.1. Bitter Peptide Database

In bioinformatics research, databases play a crucial role. They provide vast data resources and offer tools for researchers to analyze and mine information, advancing scientific progress [119,120,121]. With more bitter peptides being discovered, researchers have constructed several databases by collecting and summarizing the bitter peptides reported in the literature. For example, the BIOPEP-UWM database contains information on bioactive peptides, including the sequences of bitter peptides and their activity information [122]; BitterDB focuses on bitter molecules and their receptors, featuring detailed descriptions of over 1000 bitter molecules, quantitative sensory data on bitterness thresholds, and toxicity information [123]; TastePeptidesDB includes the detailed sequences and taste characteristics of taste-active peptides [124]; and the database on cheese-derived bitter peptides focuses on the sequences and characteristics of bitter peptides in cheese [125]. These databases collectively provide essential resources for identifying and characterizing bitter peptides, developing prediction models, and designing functional foods and pharmaceuticals. Through these databases, researchers can perform data analyses more efficiently, uncover new scientific discoveries, and propel the development of bioinformatics and related fields. This demonstrates the importance of databases in bioinformatics research. Specific database information is shown in Table 1.

### 3.2. Bitter Peptide Prediction Models

#### 3.2.1. Bitter Peptide Quantitative Structure–Activity Relationship Models

Quantitative structure–activity relationship (QSAR) models are methods for predicting the biological activity of compounds by analyzing their molecular structures. This approach is based on the core principle that molecules with the same structures often exhibit similar biological activities or chemical properties [126,127,128,129,130]. This concept was systematically introduced by Corwin Hansch in the 1960s and applied in his studies on benzoic acid derivatives [131].

QSAR models integrate chemical and biological information with mathematical and statistical methods to develop a system that can predict the activity of unknown compounds. The general steps for building a QSAR model include first collecting data on compounds with known biological activities and chemical structures; secondly, calculating the molecular descriptors of the compounds, such as their topological indices, electronic characteristics, and geometric properties; thirdly, standardizing these molecular descriptors and removing redundant or highly correlated descriptors; then, selecting appropriate mathematical and statistical methods, such as multiple linear regression, support vector machines, or neural networks, to establish the QSAR model; and finally, evaluating the predictive performance and stability of the model using methods like cross-validation and external validation sets. These steps constitute the complete process of building QSAR models from data collection to application and aid in systematically predicting a compound’s biological activity [132,133,134].

Currently, several studies on QSAR models for bitter peptides have been published. These studies have utilized various datasets, molecular descriptors, and modeling methods, achieving notable results (Table 2). The development of these models has improved the predictive capability for the bitterness thresholds of bitter peptides and provided a theoretical basis for the design and development of new bitter peptides.

There are significant trends in the evolution of QSAR models for bitter peptides. The first trend is that molecular descriptors have become increasingly complex and multidimensional. In early studies, Asao et al. used relatively simple descriptors, such as hydrophobicity, molecular size, and electronic properties [135]. Over time, researchers have introduced more complex descriptors, such as total hydrophobicity, residue count, and log mass values [136,137]. More recent studies, such as those by Wang et al. and Xu et al., have adopted more advanced and refined molecular modeling and alignment methods [117,138]. The second trend is that modeling methods have evolved from linear regression to nonlinear regression and machine learning techniques. Early research often used partial least squares regression (PLS) [139]. Recent years have seen the introduction of nonlinear models like support vector regression (SVR) [140], support vector machines (SVMs) [141,142,143], and artificial neural networks (ANNs) [144], significantly enhancing the models’ predictive power and stability. The third trend is model evaluation metrics seeing substantial improvements. Early models had relatively low coefficients of determination (R^2^) and root mean square errors of prediction (RMSEPs), but these metrics have significantly improved with in-depth research and improved methods. For instance, SVR models can achieve a high R^2^ (0.962) and a low RMSEP (0.123), demonstrating an excellent predictive performance. Overall, with the increasing complexity of molecular descriptors, the evolution of modeling methods, the enhancement of model evaluation metrics, and the expansion of dataset sizes, the development of bitter peptide QSAR models has shown increasingly strong predictive capabilities and application prospects.

#### 3.2.2. Bitter Peptide Classification Prediction Models

Machine learning classification models are based on assigning input data to predefined categories and are widely applied across various fields [145,146,147]. The construction of classification models generally involves the following steps: First, collect and prepare the data, ensuring their quality and representativeness; second, preprocess the data, including cleaning, normalizing, and extracting features to enhance the model’s performance; then, select an appropriate classification algorithm, such as logistic regression, support vector machines, decision trees, random forests, k-nearest neighbors (k-NN), or neural networks in deep learning, and train the model; next, evaluate the model’s performance using cross-validation or an independent validation set, using metrics including accuracy (ACC), sensitivity (Sn), specificity (Sp), matthews correlation coefficient (MCC), and area under the receiver operating characteristic curve (ROC); and finally, apply the trained model to classify new data, and interpret and analyze the results. Machine learning classification models excel in classification tasks by learning complex patterns and relationships from large amounts of data, which are extensively used in fields such as medical diagnosis, image recognition, natural language processing, and financial risk assessment [148,149,150,151,152,153]. Below, we detail eight different bitter peptide classifiers, their methodologies, technical features, and performances in bitter peptide prediction, as shown in Table 3.

The development of bitter peptide classifiers from 2020 to 2024 significantly reflects the evolution from traditional statistical methods to deep learning technologies (Table 3). Initially, in 2020, the iBitter-SCM primarily utilized a scoring card method (SCM) based on dipeptide propensity, which, although simple to implement, had limited capabilities in handling complex biological sequence data [154]. In 2021, with the introduction of BERT4Bitter, classifiers began utilizing a combination of BERT [155] and LSTM [156], marking a significant turning point in bitter peptide classification technology—the transition from simple statistical feature extraction to complex neural network models, allowing for deeper and more automated feature extraction [157]. By 2022 and 2023, the technology continued to lean towards ensemble and deep learning. For example, MIMML adopted meta-learning and TextCNN, emphasizing the optimization of model structure and parameters through learning how to learn better [158]. iBitter-DRLF demonstrated how to extract more complex sequence features by integrating deep learning techniques (such as SSA, UniRep, and BiLSTM) and exhibited clear advantages in enhancing model generalizability and handling diverse biological data [4]. By 2024, the development of bitter peptide classifiers reached new heights with the introduction of CPM-BP, a LightGBM-based model incorporating in-depth features [159]. This trend indicates that classification technology is moving towards greater precision and efficiency. Besides improving the accuracy and efficiency of classifiers, these advancements have also improved their adaptability to the complexity and high dimensionality of biological datasets. This implies that future bitter peptide classification technology might explore more dynamic and adaptive machine learning methods. Overall, the developmental trajectory of bitter peptide classifiers reflects the rapid progress in technology and applications with bioinformatics, showcasing the successful translation from basic research to practical applications.

**Table 3:** Bitter peptide classification prediction models.

Classifier	Dataset	Features	Algorithm	ACC	Sn	Sp	MCC	AUC	Publication	Reference
iBitter-SCM	BTP640: 320 BPs and 320 NBPs	AAC, DPC	SCM	0.844	0.844	0.844	0.866	0.904	July 2020	[154]
BERT4Bitter	BTP640: 320 BPs and 320 NBPs	Original sequence	BERT + LSTM	0.922	0.938	0.906	0.844	0.964	February 2021	[157]
iBitter-Fuse	BTP640: 320 BPs and 320 NBPs	AAC, DPC, PAAC, APAAC, AAI	SVM	0.930	0.938	0.922	0.859	0.933	August 2021	[160]
MIMML	BTP640: 320 BPs and 320 NBPs	TextCNN	Meta-learning	0.938	0.938	0.938	0.875	0.955	January 2022	[158]
iBitter-DRLF	BTP640: 320 BPs and 320 NBPs	SSA; UniRep; BiLSTM	LGBM	0.944	0.922	0.977	0.899	0.977	July 2022	[4]
Bitter-RF	BTP640: 320 BPs and 320 NBPs	AAC, TPAAC, APAAC, ASDC, DPC, DDE, GAA, GDPC, SOCNumber, QSOrder1	RF	0.940	0.940	0.940	0.890	0.980	January 2023	[161]
Umami_YYDS	129 BPs and 84 NBPs	278 descriptor features	GTB	0.896	0.917	0.875	0.792	0.980	March 2023	[124]
CPM-BP	BTP720: 360 BPs and 360 NBPs	Q, Q1, Q2, Q3, Q4, AH, N, C, Percentage-HAA, N-basic AA, LFIYWV-C, Percentage-FWY, P-X-C, RP	LightGBM	0.903	0.891	-	0.816	0.905	February 2024	[159]

Note: AAC: amino acid composition; DPC: dipeptide composition; PAAC: pseudo amino acid composition; APAAC: amphiphilic pseudo amino acid composition; AAI: Amino Acid Index; CNN: convolutional neural network; SSA: secondary structure assignment; UniRep: universal representation of protein sequences; BiLSTM: bidirectional long short-term memory; TPAAC: traditional pseudo amino acid composition; ASDC: adaptive skip dinucleotide composition; DDE: dipeptide deviation from expected mean; GAA: grouped amino acid composition; GDPC: grouped dipeptide composition; SOCNumber: sequence-order-coupling number; QSOrder1: quasi-sequence-order; Q: average hydrophobicity of peptides; Q1: percentage of the amino acids with value < 0 in peptides; Q2: percentage of the amino acids with value in range 0–1000 in peptides; Q3: percentage of the amino acids with value in range 1000–2000 in peptides; Q4: percentage of the amino acids with value in range 2000–3000 in peptides; AH: average hydrophobicity of peptides; N: hydrophobicity of amino acids located in the N-terminal of peptides; C: hydrophobicity of amino acids located in the C-terminal of peptides; Percentage-HAA: percentage of bitter-contributing amino acids (Ala, Phe, Gly, Ile, Leu, Met, Pro, Val, Tyr, and Trp) in peptides; N-basic AA: amino acids located in N-terminal of peptides were basic amino acids or not; LFIYWV-C: amino acids located in C-terminal of peptides were six kinds bitter-contributing amino acids (Leu, Phe, Ile, Tyr, Trp, and Val) or not; Percentage-FWY: percentage of three kinds of bitter-contributing amino acids (Phe, Trp, and Tyr) in peptides; P-X-C: amino acid P located in the second place from C-terminal of peptides or not; RP: adjacent RP in peptides or not; SCM: scoring card method; BERT: bidirectional encoder representations from transformers; LSTM: long short-term memory; LGBM: light gradient boosting machine; RF: random forest; GTB: gradient boosting; LightGBM: light gradient boosting machine.

The six classifiers built on the same dataset, BTP640, primarily employ machine learning or deep learning techniques. In traditional machine learning methods, features such as AAC and TPAAC, as mentioned in the table above, are manually extracted, chosen based on extensive experimental validation and varying in performance depending on the application scenario [124,161]. Some feature extraction methods might adversely affect classification effectiveness, requiring researchers to manually select and adjust these methods based on specific problems and perform feature screening to ensure that the chosen features achieve the optimal classifier performance [162,163,164]. Deep learning is superior for its performance and ability to implement an “end-to-end” processing workflow, meaning that users need to provide sequences to the deep learning model that can automatically perform feature extraction, model construction, and feature selection, outputting the results. Deep learning models continually iterate to optimize parameters, automatically constructing the best-suited model without the need for tedious manual settings like traditional machine learning. This increased level of automation significantly simplifies the model development process, demonstrating substantial advantages when handling complex biological data [165,166].

Overall, the progress of these models showcases the evolution from manual feature engineering to automated deep learning, reflecting the ongoing innovation and development in bitter peptide classification technology. This development has enhanced the performance and efficiency of classifiers and provides more powerful tools for future bioinformatics research.

#### 3.2.3. Shift in Research Directions

Considering the two types of models, the research focus has shifted over time. Before 2020, researchers primarily focused on constructing QSAR models to study the bitterness thresholds of bitter peptides. After 2020, the direction shifted towards developing classification prediction models for determining whether a peptide possesses bitterness.

Several factors may be responsible for this change in research directions and goals. (1) Before 2020, the lack of dataset size and quality led researchers to focus more on quantifying the bitterness threshold for each bitter peptide. Over time, with more experimental data being accumulated, datasets increased in size and diversity, allowing researchers to better construct and validate classification models [167]. (2) Rapid advancements in machine learning and artificial intelligence technologies provided more tools and methods for classification models, which can handle large-scale data more efficiently and excel in dealing with nonlinear and complex relationships, enabling researchers to use these advanced technologies to more accurately predict and classify bitter peptides [168]. (3) The food industry and health sector increasingly demand the rapid identification of bitter peptides. Compared to merely quantifying its bitterness threshold, quickly determining whether a peptide is bitter is more practical for real-world applications, such as discovering new food additives, where the rapid screening of bitter peptides can significantly enhance development efficiency [169,170]. (4) Classification models generally outperform regression models in terms of computational and prediction efficiency, which can more quickly predict and classify bitter peptides from candidate sequences for industrial production and scientific research [171]. As research progresses, scientists are increasingly recognizing that understanding the classification attributes of bitter peptides (whether they are bitter peptides) is often more crucial than quantitatively analyzing their bitterness thresholds. Classification models can help to reveal the characteristic patterns of bitter peptides, guiding the design of peptides with specific or no bitterness characteristics.

## 4. Future Directions

### 4.1. Database Enhancement

Databases focused on bitter peptides encompass extensive sequence data and activity information, lacking comprehensive details about sources and functions. They will be updated and elevated with professional properties [122,123,124]. To expand the current databases, we can consider the following three aspects. (1) Diverse Data Sources: In addition to the bitter peptides produced during fermentation processes, it is also important to collect bitter peptides from other sources such as plant proteins and marine organisms to enrich the diversity of databases. (2) Integration of Multimodal Data: We should not only collect the sequences of bitter peptides, but also include their bitterness thresholds, structural information, important site details, and biological activity characteristics. (3) Establishment of a Regular Update System: A regular update should promptly incorporate the latest research findings and discoveries of bitter peptides to ensure that databases remain cutting-edge and practical. The above approach aligns with the way many established protein databases, such as the Protein Data Bank (PDB) and universal protein resource (UniProt), were constructed and are maintained [172,173].

Since bitter peptides have applications in the food and pharmaceutical industries, there is a need for the development of more specific databases. For example, the database of cheese-derived bitter peptides specifically includes bitter peptides produced during cheese fermentation [125]. The methods and maintenance strategies for building a specialized database for specific fields and a comprehensive bitter peptide database are fundamentally similar, with the primary difference being the focus of the information collected. For instance, a specialized database could be developed for bitter peptides in food science, emphasizing factors such as the sources of bitter peptides, their bitterness thresholds, their impacts on food taste, and their potential health benefits. Additionally, a separate database could be dedicated to bitter peptides with potential antidiabetic effects, concentrating on their therapeutic efficacy, specific mechanisms of action, and related data. These specialized databases would play a crucial role in food safety and disease research, facilitating researchers to efficiently utilize bitter peptides for various studies and practical applications.

### 4.2. Diversity of Models

#### 4.2.1. Classification Models

Classification models for bitter peptides have achieved significant results in predicting their presence. However, due to the small datasets used to train these models, their generalization performance may not be ideal, limiting their effectiveness in practical applications. Future objectives should promote the robustness and practicality of these models and focus on several areas. (1) Building more comprehensive datasets is crucial for improving model generalization. Larger datasets provide more training samples to enhance a model’s learning capability and cover a diverse range of bitter peptide sequences and features, ensuring a more stable performance with different data types. Additionally, increasing the diversity in datasets, including bitter peptides from other sources and with other functions, will enable models to better adapt to various application scenarios [174,175,176]. (2) Employing more complex and advanced algorithms to construct classification models will enhance their performance. Existing models primarily rely on traditional machine learning methods and the initial applications of deep learning techniques. In the future, more cutting-edge deep learning architectures, such as the Transformer model and its variants, which excel in handling high-dimensional and complex feature data, could be beneficial. By integrating the advantages of multiple algorithms, more precise and robust classification models can be constructed [177,178,179]. (3) The robustness and interpretability of models are crucial for future research. In addition to improving prediction accuracy, it is essential to focus on the stability of models across different datasets and environments to ensure their reliability in practical applications. Furthermore, enhancing the interpretability of models, allowing researchers to understand the decision-making processes of these models, is crucial for advancing the depth of bitter peptide research [180,181,182].

#### 4.2.2. Interaction Models

There are 25 bitter taste receptors with different structures and ligands in the human body, capable of binding distinct bitter peptides. Bitter taste receptors have a high level of specificity when binding to bitter peptides, meaning that not a single receptor can bind to all types of bitter peptides [183,184]. Physiological responses, including lowering blood pressure and the stimulation of gastrointestinal motility, are only triggered when a bitter peptide binds to its specific receptor. This underscores the interactions between bitter peptides and their specificity receptors. In this relationship, bitter receptors act as drug targets, with the bitter peptides serving as the drugs, effective only when the drug binds to its target [185,186]. In bioinformatics, there has been considerable research on protein and peptide interactions, such as studying protein–ligand binding characteristics through molecular docking and molecular dynamics simulations [187,188,189].

Thus, developing a model to predict the interactions between bitter peptides and receptors is crucial for future research. This model would help us better understand the interactions between bitter peptides and receptors and provide robust support for the development of targeted drugs. By constructing such a model, researchers can identify bitter peptides with specific physiological effects and design new pharmaceuticals or functional foods. In practice, developing these predictive models can utilize various technical approaches. For example, machine learning and deep learning algorithms can be employed in training with a vast amount of known data on bitter peptide–receptor bindings to build highly accurate prediction models [190,191,192,193]. Combining computational methods like molecular docking and molecular dynamics simulations can further validate and optimize prediction outcomes [194,195]. The integration of these methods will aid in developing more precise and reliable predictive models.

#### 4.2.3. Generative Models

The concept of using generative models for simulating simple data distributions dates back to the 1950s [196]. However, it was not until the early 21st century, with significant improvements in computational power and the advent of the big data era, that generative models began to evolve and rapidly demonstrate their formidable potential. In recent years, generative models have made breakthroughs in many fields, such as the large language models exemplified by OpenAI’s GPT-4, which can generate text nearly indistinguishable from that produced by humans [197].

In bioinformatics, generative models have also played a significant role. DeepMind’s AlphaFold model series is a milestone example, accurately predicting the three-dimensional structures of proteins through machine learning methods, advancing biomedical research and drug development. The successful application of AlphaFold demonstrates the potential of generative models in predicting complex biological structures [198,199]. The latest de novo protein generative models, such as ProGen [200] and ProteinGAN [201], can produce entirely new proteins not yet found in nature, providing new ideas and insights for protein design [202,203,204].

Constructing a specialized bitter peptide generative model is of tremendous application value, which could specifically provide the properties of bitter peptides, such as their solubility and stability, or combine two peptides with different functions to create multifunctional effects [205]. Further, it could generate bitter peptides with entirely new functions, opening up new research avenues. Developing bitter peptide generative models would be achieved in the following directions. Firstly, collecting and organizing more bitter peptide sequences and their related property data to establish a rich dataset. Secondly, employing advanced generative model algorithms, such as Generative Adversarial Networks (GANs) and Transformers, for model training and optimization [206,207,208]. Finally, although a comprehensive evaluation system to assess the quality of newly generated bitter peptides from generative models has yet to be established, several methods are currently available for this purpose, as follows: (1) utilizing structural prediction tools (such as AlphaFold and ESM Fold) to predict the structures of generated bitter peptides and comparing them with known bitter peptides to evaluate their structural similarity and potential functionality; (2) using tools (such as foldX and RoseTTA) to calculate the free energy and stability of the generated peptides, with lower-energy conformations typically being more stable, which helps to assess their bioactivity and stability; (3) employing molecular docking methods (such as AutoDock Vina and Dock) to study the interactions between the generated bitter peptides and specific receptors, identifying potential binding sites and affinities, thereby further supporting functional predictions; and (4) when feasible, cloning the genes of the generated bitter peptides into expression vectors and conducting in vitro or cell-based experiments to verify their actual functions, assessing whether they exhibit similar or superior characteristics compared to natural bitter peptides. We expect that bitter peptide generative models might accelerate research and applications that can provide new tools and methods for food science and pharmaceutical development.

### 4.3. Experimental Evaluation

Experimental validation has always been a crucial part of bioinformatics, serving as the best standard for assessing various model performances [209]. Accuracy and robustness can be evaluated in classification models using data from newly identified bitter and non-bitter peptides [149], while the validation process for generative models is more complex. Initially, the generated bitter peptides can be screened based on their physicochemical properties, such as their stability and solubility. Subsequently, these peptides are necessary to confirm their biological activity and functionality [200,210,211,212]. However, there is a lack of quantitative metrics to determine whether generated bitter peptides are superior to those in nature. Comparing the activity of bitter peptides under the same or optimized conditions may yield different conclusions [213,214]. Therefore, setting fair and reasonable evaluation standards is an issue that needs in-depth exploration.

Despite the current uncertainties in model validation structures, this remains an essential path for bioinformatics research. Future studies should continuously optimize experimental evaluation methods and establish a more scientific and systematic assessment system to more accurately gauge the merits of generative models. Such progress will aid in the development and applications of bioinformatics models and help to advance the field.

## 5. Conclusions

Bitter peptides are small molecular peptides produced during the hydrolysis of proteins, commonly found in everyday foods such as meats and dairy products. Their bitterness is primarily caused by the exposed hydrophobic amino acids within the peptides, which bind to G-protein-coupled bitter taste receptors, triggering complex signal transduction and ultimately forming the perception of bitterness in the brain. Apart from being extensively studied in food science for their taste characteristics, bitter peptides are also of significant research interest due to their various biological activities. Studies have shown that bitter peptides possess multiple health benefits, including antihypertensive, antidiabetic, antioxidant, antibacterial, and immune regulation properties, thus displaying broad application prospects in the biomedical field. Particularly in managing blood pressure and diabetes, bitter peptides have shown significant therapeutic potential. Therefore, research on bitter peptides enhances our understanding of the causes and regulation of food bitterness and provides a scientific basis for developing new therapies and improving public health. With advances in food science and biotechnology, the multifunctionality and potential applications of bitter peptides will become a valuable area of research.

We provide a detailed introduction to the research on bitter peptides in the field of bioinformatics, covering several aspects: (1) bitter peptide databases, which are crucial for research on bitter peptides; (2) QSAR models predicting the bitterness levels of peptides, characterizing bitter peptides using molecular descriptors, and employing simple regression algorithms to predict bitterness thresholds, which are important for studying the bitterness of peptides and for debittering processes in the food industry; and (3) predictive models for bitter peptides, extracting features through various means and building models with machine learning algorithms to predict whether a peptide is a bitter peptide, offering significant value to experimental researchers and food industry professionals. Despite significant progress in research on bitter peptides through bioinformatics, several challenges remain, including incomplete data, leading to a lack of diversity and limiting the comprehensiveness of databases, and the need for models with improved generalizability and robustness, especially with small sample datasets. Additionally, the complexity of the interactions between bitter peptides and taste receptors increases the difficulty in developing predictive models. The accuracy and efficacy of generative models urgently require a more scientific evaluation system. These challenges must be addressed through the continuous optimization of data collection, model construction, and experimental validation methods to advance bitter peptide research towards application-oriented directions.

Future research directions in bitter peptide studies primarily include four areas. First, enhancing bitter peptide databases, particularly developing specialized databases targeted at different application domains to improve research and application efficiency. Second, developing models with an advanced performance and interpretability by building larger datasets and employing advanced algorithms that can predict the interactions between bitter peptides and taste receptors to advance new drug development and functional food design. Third, exploring the application of generative models in bitter peptide research to produce peptides with specific or entirely new functions, which should accelerate research and applications. Finally, experimental evaluation will be crucial in validating model performance and establishing a scientific and systematic assessment system to propel the development of bioinformatics and biotechnology.

## Figures and Tables

**Table 1:** Bitter peptides related database.

Database	Data Volume	Link	Publication	Main Purpose	References
BIOPEP-UWM	2275 BPs	https://biochemia.uwm.edu.pl/biopep-uwm/ (accessed on 11 September 2024)	2019	Widely used in the design of functional foods and research on bioactive peptides.	[122]
Bitter DB	1041 BMs	https://bitterdb.agri.huji.ac.il/dbbitter.php (accessed on 11 September 2024)	2019	Extensively used in selecting experimental ligands and developing bitter taste prediction models.	[123]
Database of Cheese-Derived Bitter Peptides	226 BPs	https://github.com/Kuhfeldrf/A-comprehensive-database-of-cheese-derived-bitter-peptides (accessed on 11 September 2024)	2023	Aids in understanding the sensory properties of cheese and quality control.	[125]
TastePeptidesDB	787 BPs	http://tastepeptides-meta.com/TastePeptidesDB (accessed on 11 September 2024)	2024	Helps with the identification and characterization of taste-active peptides in foods.	[124]

Note: BP: bitter peptide; BM: bitter molecule.

**Table 2:** Bitter-peptide-related QSAR models.

Model	Dataset	Molecular Descriptors	Q^2 a^	R^2 b^	RMSEP ^c^	Publication	Reference
PLS	48 bitter dipeptides	Hydrophobicity, molecular size/volume, and electronic properties	-	-	-	November 1987	[135]
PLS	229 bitter peptides	Total hydrophobicity, residue number, and log mass values	-	Dipeptides 0.750 Pentapeptide 0.900	Dipeptides 0.530 Pentapeptide 0.480	November 2006	[136]
SVR	48 bitter dipeptides	5 molecular descriptors ^d^	0.912	0.962	0.123	May 2010	[137]
MLR SVM ANN	229 bitter peptides	20 molecular descriptors ^e^	-	MLR 0.723 SVM 0.739 ANN 0.767	-	November 2013	[118]
CoMFA, CoMSIA	52 bitter peptides	Molecular modeling and molecular alignment	COMFA0.534 COMSIA0.547	COMFA0.716 COMSIA0.579	COMFA0.430 COMSIA0.423	January 2019	[138]
PLS	48 dipeptides, 52 tripeptides, and 23 tetrapeptides	14 molecular descriptors ^f^	Dipeptides 0.941 ± 0.001 Tripeptides 0.742 ± 0.004 Tetrapeptides 0.956 ± 0.002	Dipeptides 0.950 ± 0.002 Tripeptides 0.770 ± 0.006 Tetrapeptides 0.972 ± 0.002	Dipeptides 0.139 ± 0.002 Tripeptides 0.282 ± 0.004 Tetrapeptides 0.127 ± 0.004	August 2019	[117]

Note: PLS: partial least squares regression; SVR: support vector regression; MLR: multiple linear regression; SVM: support vector machine; ANN: artificial neural network; CoMFA: comparative molecular field analysis; and CoMSIA: comparative molecular similarity indices analysis. ^a^ Q^2^ (cross-validated coefficient of determination): Q^2^ is the coefficient of determination obtained through cross-validation. It is used to assess the predictive ability and robustness of the model. The higher the Q^2^ value, the better the model performs during cross-validation, indicating stronger predictive power. ^b^ R^2^ (coefficient of determination): R^2^ reflects the degree of fit of the model to the training data, ranging from 0 to 1. The closer the R^2^ value is to 1, the stronger the model’s explanatory power for the training data. However, a high R^2^ value does not necessarily indicate good predictive ability, as it may suggest overfitting to the training data. ^c^ RMSEP (root mean square error of prediction): RMSEP is used to measure the prediction error of the model on the test set. The smaller the value, the better the model’s performance. ^d^ 5 molecular descriptors: largely hydrophobicity, steric properties or side chain bulk/molecular size, preferences for amino acids to occur in α-helices, composition, and the net charge. ^e^ 20 molecular descriptors: 2D autocorrelation, 3D-MoRSE descriptors, centered fragments, Burden eigenvalues, connectivity indices, constitutional descriptors, edge adjacency indices, eigenvalue-based indices, functional group counts, geometrical descriptors, GETAWAY descriptors, information indices, molecular properties, Randic molecular profiles, RDF descriptors, topological charge indices, topological descriptors, walk and path counts, and WHIM descriptors. ^f^ 14 molecular descriptors: 3z-scale, 5z-scale, DPPS (Divided Physiochemical Property Scores), MS-WHIM-extended (Weighted Holistic Invariant Molecular approach applied on Molecular Surface), ISA-ECI (Isotropic Surface Area and Electronic Charge Index), VHSE (Principle Components Score Vectors of Hydrophobic, Steric, and Electronic Properties), FASGAI (Factor Analysis Scale of Generalized Amino Acid Information), VSW (Vector of Principle Components Scores for Weighted Holistic Invariant Molecular Index), T (Topological)-scale, ST (Structural Topological)-scale, E-scale, V, G-scale, and HESH (Hydrophobic, Electronic, Steric, and Hydrogen).

## Data Availability

Not applicable.
